# Chronic Lymphocytic Inflammation with Pontine Perivascular Enhancement Responsive to Steroids (CLIPPERS Syndrome): A Case Report and Literature Review

**DOI:** 10.1155/2023/5811243

**Published:** 2023-07-21

**Authors:** Nripesh Man Shrestha, Niranjan Acharya, Rabindra Desar

**Affiliations:** Civil Service Hospital, Kathmandu, Nepal

## Abstract

CLIPPERS is a rare, chronic inflammatory neurological syndrome affecting multiple regions of the brain including the brainstem, cerebellum, and spinal cord. More than 100 cases have been documented globally since its initial description in 2010. This article reports the first case of the CLIPPERS syndrome in Nepal. Clinical and radiological evidences of the patient lead to the diagnosis of this disease. Brain MRI reveals punctate and curvilinear gadolinium enhancement in the pons and cerebellum, which is diagnostic for the disease. Steroid therapy has been reported to be effective in treating CLIPPERS syndrome. Although its pathophysiology indicates an immune-mediated process, the etiology is yet unknown. The treatment and prognosis of this illness depend on an early and accurate diagnosis.

## 1. Introduction

Chronic lymphocytic inflammation with pontine perivascular enhancement responsive to steroids is referred to as CLIPPERS. It is an unidentified neurological illness that is steroid sensitive. Lymphocyte infiltration mostly affects the perivascular regions of the pons, midbrain, and cerebellum. Its clinical presentation varies depending on where the lesion is, although gait ataxia and dysarthria are most common. Since the patients' unusual presentations and hazy neurological symptoms can be confusing to the physician, MRI plays a larger role in the diagnosis than a straightforward neurological examination [1]. The usual pepper-like speckles seen on the brain MRI are accompanied by curvilinear enhancement in the brainstem, cerebellum, and spinal cord. Steroid hormones can be used to treat CLIPPERS successfully [2]. However, trials and case studies have mentioned the use of steroid-sparing agents such as azathioprine and hydroxychloroquine that successfully keep the patients on remission. Recently, even antitubercular agents and immunotherapy have been under trial for treatment of this disease [3]. Here, we report a case of clinically and radiologically diagnosed CLIPPERS syndrome treated with steroids and kept on the maintenance therapy with azathioprine and hydroxychloroquine with successful remission till date.

## 2. Case Report

The case in Nepal is of a 26-year-old female who presented to our center with the complaints of difficulty in walking and dizziness for the past 2 weeks. During this period, she had progressively noticed difficulty while walking and felt heaviness in her body, resulting in inability to walk in a straight line. Dizziness manifested as subjective sensation of imbalance of the body, relieved by lying down on bed and occasionally associated with nausea. The symptoms had insidious onset and were not preceded by any sort of illness. She did not have any history of previous hospitalization and no known comorbidities or any genetic or environmental risk factors that would precipitate this condition.

On presentation to our center, her pulse was 92 b/min; blood pressure 100/60 mmHg; and temperature 97°F. On neurological examination, she had mild degree left-sided lateral rectus palsy, wide-based ataxic gait, unable-to-perform tandem walk, and Romberg's sign positive. All other systemic examinations were normal.

**Figure 1:**
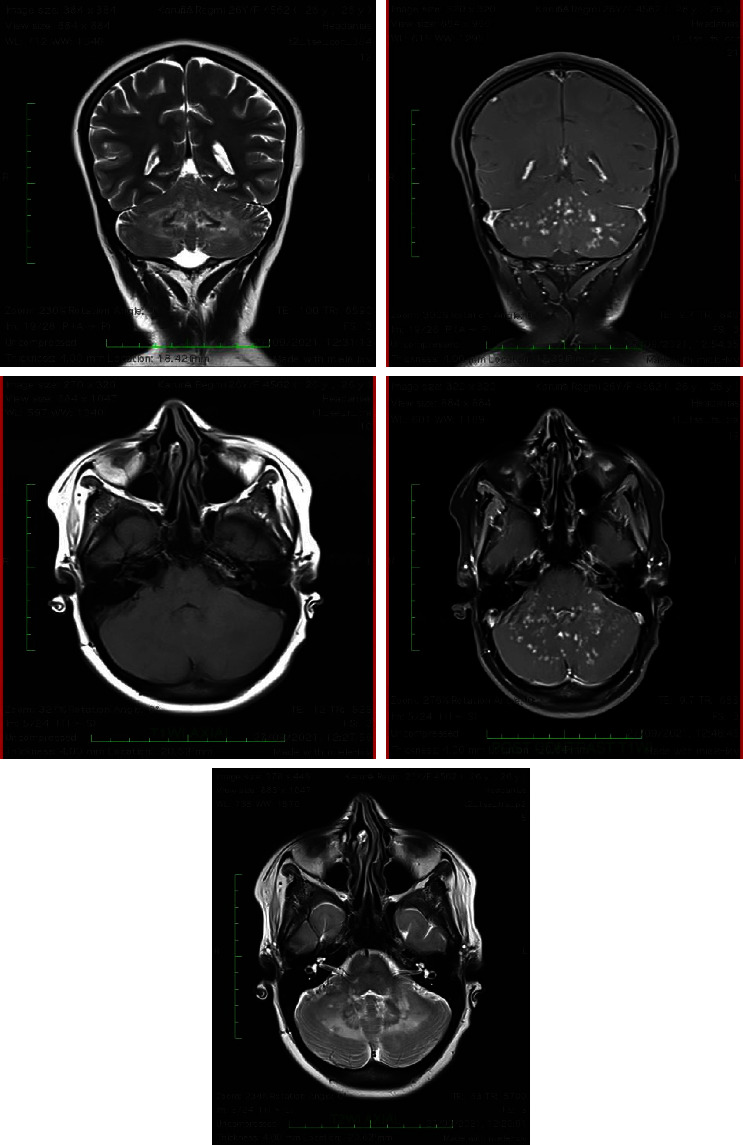
MRI brain images of the patient with CLIPPERS syndrome showing diffuse and patchy altered high T2/FLAIR signal intensity areas with multiple punctate, patchy, linear enhancement in bilateral middle cerebellar peduncles, adjacent bilateral cerebellum, vermis and pons, and left parietal lobe.

Brain MRI scans of the patient (Figure 1) revealed diffuse and patchy altered high T2/FLAIR signal intensity areas with multiple punctate, patchy, linear enhancement in bilateral middle cerebellar peduncles, adjacent bilateral cerebellum, vermis and pons, and left parietal lobe in the postcontrast images.

Her routine blood tests were within normal limits. Autoimmune workup (ANA, p-ANCA, and c-ANCA) was normal. Then, CSF analysis was carried out which showed high protein and sugar contents. Oligoclonal IgG bands and IgG index were negative.

**Figure 2:**
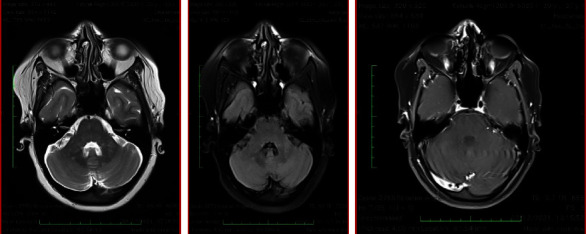
MRI brain images of the same patient, post-treatment (methylprednisolone, azathioprine, and hydroxychloroquine), showing resolution of the lesions in comparison to the previous MRI films.

The patient was treated with IV methylprednisolone for 5 days. She showed significant improvement following 2 doses of IV steroids. Her neurological examination was normal after 5 days of IV steroids. She was discharged with oral steroids, steroid-sparing agents (azathioprine and hydroxychloroquine), and other supportive therapies. She was called for follow up after 1 month for a brain MRI (Figure 2). The imaging revealed resolution of the lesions in comparison with the previous scans.

The patient has been on follow-up since then and is on remission under hydroxychloroquine and azathioprine.

## 3. Discussion and Literature Review

CLIPPERS syndrome is a rare chronic inflammatory disease of the central nervous system that was first reported in 2010 [2]. Because of its relatively recent discovery and rare pathological data, all reported cases have been sporadic. Therefore, the etiology and pathogenesis of the disease remain unclear. The disease can occur in all age groups, and both men and women can be affected. In the cases that have been recorded so far, there have been somewhat more male patients than female patients, and the sickness has primarily affected people in their 20s and 30s, with a few cases involving children. CLIPPERS pathogenesis is largely unknown, and its clinical manifestations are polymorphic and sometimes confounding. Patients tend to have subacute onset and progressive aggravation [4]. The main lesions are in the pons, but CLIPPERS pathology can also involve other parts of the brainstem, corpus callosum, white matter of the cerebellar and basal ganglia areas, and spinal cord. The main clinical manifestations are ataxia, dysarthria, diplopia, and sensory disorders and can include a combination of different symptoms according to the location of the lesion. There can be a variety of atypical presentations and the clinical scenario can be often misleading; thus, a predominant role in the diagnosis is played by MRI rather than a neurological examination [1]. Due to the adversity and complexity in the diagnosis of CLIPPERS, Tobin et al. proposed the following diagnostic criteria [5]:

(1) Clinical
  - Subacute pontocerebellar dysfunction, with or without other CNS symptoms such as cognitive dysfunction and myelopathy
  - CNS symptoms responsive to corticosteroid therapy
  - Absence of peripheral nervous system disease
  - Lack of alternative better explanation for clinical presentation
(2) MRI
  - Homogenous, gadolinium enhancing nodules without ring enhancement or mass effect predominating in the pons and cerebellum, measuring <3 mm in diameter
  - Marked improvement in abnormal gadolinium enhancement with corticosteroid treatment
  - Homogenous T_2_ signal abnormality where degree of T_2_ does not significantly exceed the size of the area of postgadolinium enhancement
  - Spinal cord lesions with similar T_2_ and gadolinium enhancing lesions as abovementioned.
(3) Neuropathology
  - Dense lymphocytic inflammation with perivascular predominance and parenchymal diffuse infiltration; both white matter and gray matter could be involved
  - T cells predominating infiltration (CD4 > CD8) with variable macrophage components
  - Absence of myelin loss or focal secondary myelin loss
  - Lack of alternative better explanation for pathological presentation
Definite CLIPPERS: patient fulfilling all clinical, radiological criteria and neuropathological criteria
Probable CLIPPERS: patients fulfilling all clinical and radiological criteria without available neuropathology [5]

So far, the etiology of CLIPPERS syndrome remains unknown. Mélé et al. demonstrated that CLIPPERS syndrome is an autoimmune disease that is mediated by T helper 17 cells [6]. Its pathogenesis supports this opinion and suggests a possible symptomatic lymphohistiocytic immune reaction. Histopathologically, CLIPPERS is an inflammatory lesion with the infiltration of lymphocytes involving both the white and gray matter.

The imaging diagnosis of CLIPPERS syndrome is mainly performed using enhanced MRI brain scans. The imaging manifestations are multiple lesions in the pons, midbrain, and cerebellum, as thin curvilinear high signal enhancement shadows, presenting pepper-like enhancement. There can be various autoimmune diseases (such as autoimmune encephalitis, Bickerstaff encephalitis, and CNS vasculitis) and inflammatory demyelinating diseases (such as ADEM, MS, and NMO) or even CNS infections and paraneoplastic syndromes that can mimic the CLIPPERS syndrome and have similar presentations. Therefore, following the diagnostic criteria proposed by Tobin et al., if patients fulfill all clinical, radiological, and pathological criteria, only then the term “definite CLIPPERS” can be applied. While it is ideal that neuropathology be examined in suspected CLIPPERS, given the eloquent CNS regions primarily affected, this will not be feasible in a substantial number of cases. Therefore, for patients fulfilling clinical and radiological criteria but without neuropathological examination, the term “probable CLIPPERS” is proposed.

The age of the current patient was 26 years old, and the clinical manifestations were difficulty in walking and dizziness, without specific indications in laboratory examinations. MRI revealed that the brainstem, cerebellum, and spinal cord were speckled with abnormal signal shadows. After enhancement, these regions presented “pepper-like” irregular reinforcement. Steroid therapy was effective. On the whole, the patient confirmed to the diagnosis of CLIPPERS syndrome.

The mainstay of treatment for CLIPPERS syndrome is a large dose of corticosteroids [4, 7]. Brain MRI enhancements of patients with CLIPPERS syndrome before and after treatment have been compared and show the characteristic signs of pepper-like appearance disappeared after treatment in many cases. However, no clinical studies have shown that this disease can be cured; so, long-term steroid maintenance therapy is needed to prevent disease recurrence. Due to the wide variety of adverse effects of the steroid therapy, alternative therapies (steroid-sparing agents) are on trial in various subjects. Some trials are showing effectivity of immunotherapy by CD20 monoclonal antibodies, rituximab, in maintaining remission [8]. Some reports also mention the effective use of tocilizumab [7]. In addition, the use of steroid-sparing agents such as hydroxychloroquine, azathioprine, methotrexate, and cyclophosphamide shows clear evidence of its effectiveness. The patient under treatment with steroid-sparing agents had to be on remission for a period of >24 months for the effectiveness of the agent to be labeled as “probable.” The corticosteroid-sparing agents whose efficiency is “probable” are methotrexate in four cases, cyclophosphamide in two cases, and hydroxychloroquine in one case [7]. Considering the risk benefit ratio of corticosteroid-sparing agents, methotrexate seems to be the most suitable. These agents not only lead to rapid remission but also effectively prevent recurrence with less adverse reactions.

CLIPPERS syndrome seems to be associated with hepatitis B mostly, and for these patients, the treatment is similar with addition in the antiviral therapy in appropriate cases [9]. Other case reports also mention the association of CLIPPERS with CMV infection, MS, lymphoma, and HLH, with the treatment strategies being adapted according to the associated disease.

In conclusion, the subject under treatment has apparently been benefitted and is on remission with the use of azathioprine and hydroxychloroquine. Her MRI scans also showed the resolution of the previous lesions and the patient is in normal condition on follow-up.

However, CLIPPERS is a disease yet to be explored due to its rarity. In order to mitigate this disease, many case series have to be reported and different trials have to be carried out all around the world to formulate the specific treatment options for the disease.

## Data Availability

The data used to support this study are available from the corresponding author upon request.

## Abbreviations

CLIPPERS: Chronic lymphocytic inflammation with pontine perivascular enhancement responsive to steroids
CNS: Central nervous system
MRI: Magnetic resonance imaging
CT: Computed tomography
CSF: Cerebrospinal fluid
PCGE: Punctate and curvilinear gadolinium enhancing lesions
MS: Multiple sclerosis
HLH: Hemophagocytic lymphohistiocytosis
ADEM: Acute demyelinating encephalomyelitis
NMO: Neuromyelitis optica.
